# Maternal mortality

**Published:** 2011-02

**Authors:** J Moodley

**Affiliations:** For the National Committee on Confidential Enquiries into Maternal Deaths, National Department of Health, South Africa; Women’s Health and HIV Research Group, Nelson R Mandela School of Medicine, University of KwaZulu-Natal, South Africa

Issues surrounding maternal mortality have recently been widely published in both the lay media and the health fraternity literature. Possible reasons for this are that there are only five years remaining until the 2015 deadline to have achieved the United Nations Millennium Development Goals (MDGs). The general impression among health professionals is that there has been slow progress in achieving MDG 5 (maternal health), which targets a three-quarters reduction in maternal deaths from 1990–2015. There have, however, been two recent publications, which are reflective of slow but significant progress in the reduction of maternal mortality in both high- and low-income countries.^1^,^2^

Last year, Hogan *et al.*, using sophisticated mathematical models, estimated a total of 342 900 maternal deaths for 2008 in 181 countries, and a 1.8% rate of annual decline in mortality between 1990 and 2008. The authors showed a decline in maternal mortality ratios (MMRs) in both high- and low-income countries, except for some countries in sub-Saharan Africa and Asia.^1^

In the latter part of 2010, the World Health Organisation, the United Nations Population Fund, the United Nations Children’s Fund and the World Bank issued the latest estimates on global MMRs. According to these estimates presented for 171 countries, approximately 358 000 deaths occurred worldwide in 2008.^2^ The global maternal mortality ratio fell by 34%, with the biggest reductions occurring in eastern Asia and northern Africa (63 and 59%, respectively). It should be noted however, that the levels and trends varied widely within regions. Although there was a decline in some African countries, South Africa, Botswana, Swaziland, Kenya and Zimbabwe were estimated to have increased MMRs. The possible reason for the lack of reduction or increase in MMRs in these countries is probably the impact of HIV/AIDS.

Overall, it was estimated that in 2008 there were 42 000 deaths among pregnant women due to HIV/AIDS.^2^ In South Africa, the Saving Mothers report 2005–2007 indicated that non-pregnancy infections (mainly HIV/AIDS) are the commonest causes of maternal mortality.^3^ The other major causes of maternal deaths in South Africa are shown in Table 1. Hypertensive disorders of pregnancy are the commonest direct causes of maternal mortality. Poorly controlled pre-eclampsia (both in the intra- and postpartum periods) are common avoidable factors.

**Table 1.** Primary Obstetric Causes Of Maternal Deaths

	*2005–2007*	
*Primary Obstetric Cause*	*N*	*%*
Direct	1819	45.9
Hypertension	622	15.7
Postpartum haemorrhage	383	9.7
Antepartum haemorrhage	108	2.7
Ectopic pregnancy	55	1.4
Abortion	136	3.4
Pregnancy-related sepsis	223	5.6
Anaesthetic related	107	2.7
Embolism	57	1.4
Acute collapse	128	3.2
Indirect	1966	49.7
Non-pregnancy-related infections	1729	43.7
AIDS	915	23.1
Pre-existing maternal disease	237	6.0
Unknown	174	4.4
Coincidental	118	

Besides HIV/AIDS, hypertensive disorders and obstetric haemorrhage, which are major causes of maternal deaths, some mothers are diagnosed to have medical conditions such as cardiac disease for the first time only in pregnancy. Detection of cardiac disease, cardiomyopathy, control of hypertension and diabetes, with proper advice on family planning, may help decrease mortality and mortality associated with medical conditions in pregnancy.

Maternal mortality is also impacted on by racial disparities. Bryant *et al*. have shown that African-American mothers have a three- to four-fold higher mortality than other major racial or ethnic groups in the USA, and propose a five-domain framework to identify contributors, namely, behaviour patterns, genetic predisposition, social circumstances, environmental exposures and shortfalls in medical care. They found that social circumstances and medical care were the most important factors.^4^

Therefore, interventions to reduce maternal mortality must address social determinants of health besides focusing on reducing obstetric haemorrhage by improving care in labour and delivery, reducing venous thrombo-embolism with DVT thrombophylaxis, reducing deaths from cardiac disease with education for earlier recognition, and effective multi-disciplinary care. Access to care, particularly longitudinal care for women with underlying medical conditions, is a critical issue and should become a part of the care provided by all working in the field of cardiovascular medicine.
